# Corni Fructus Alleviates UUO-Induced Renal Fibrosis via TGF-*β*/Smad Signaling

**DOI:** 10.1155/2022/5780964

**Published:** 2022-05-06

**Authors:** Jin A. Lee, Mi-Rae Shin, Seong-Soo Roh

**Affiliations:** Department of Herbology, College of Korean Medicine, Daegu Haany University, 136, Sinchendong–ro, Suseong-gu, Daegu 42158, Republic of Korea

## Abstract

Renal fibrosis is a type of chronic kidney disease (CKD) induced by infiltration of inflammatory cells, myofibroblast accumulation, and ECM production in the kidney. From a long time ago, Corni Fructus (CF) is known to supplement the liver and kidney with its tepid properties. In this study, we investigated the renal protective mechanism of CF, which is known to supplement the kidney, in rat model of unilateral ureteral obstruction (UUO). After inducing UUO through surgery, the group was separated (*n* = 8) and the drug was administered for 2 weeks; normal rats (normal), water-treated UUO rats (control), CF 100 mg/kg-treated UUO rats (CF100), and CF 200 mg/kg-treated UUO rats (CF200). As a result of histopathological examination of kidney tissue with H&E, MT, and PAS staining, it was confirmed that the infiltration of inflammatory cells and the erosion of collagen were relatively decreased in the kidneys treated with CF. Also, CF significantly reduced the levels of MDA and BUN in serum. As a result of confirming the expression of the factors through western blotting, CF treatment significantly reduced the expression of NADPH oxidase and significantly regulated the AMPK/LKB1/NF-*κ*B pathway associated with inflammation. In addition, it downregulated the expression of major fibrotic signaling factors, such as *α*-SMA, collagen I, MMP-2, and TIMP-1, and significantly regulated the TGF-*β*1/Smad pathway, which is known as a major regulator of renal fibrosis. Taken together, these findings indicate that CF can alleviate renal fibrosis by regulating the TGF-*β*1/Smad pathway through inhibition of oxidative stress in UUO.

## 1. Background

Chronic kidney disease (CKD) is a disease that refers to when symptoms, such as abnormal kidney function and structure, decrease in function persist for more than 3 months and is recognized as a major health problem due to high medical costs and mortality [1, 2]. CKD is caused by obesity, smoking, and continuous exposure to nephrotoxic drugs and causes complications such as cardiovascular disease, diabetes, dyslipidemia, and bone and mineral disorders [2, 3]. Also, various chronic kidney diseases, such as ureteral obstruction and diabetes, can cause inflammatory cell infiltration and myofibroblast accumulation, and it induces excessive accumulation of extracellular matrix (ECM) through induction of increased ECM production and decreased degradation, leading to renal fibrosis [4, 5]. Inflammation and oxidative stress play an important role in the cause of renal fibrosis. Several studies have shown that oxidative stress is involved in the activation of transforming growth factor *β*1 (TGF-*β*1) activity, a major pathway for fibrosis [6, 7]. TGF-*β*1 signals are transmitted to the intermediate Smad, and especially, Smad2 and Smad3 are considered as major pathogenic mechanism of renal fibrosis [8]. Currently, several studies aimed at inhibiting TGF- *β*1 signaling have shown good results for the treatment of renal fibrosis, but limited advances have been made in translating this research to patients with CKD [9].

In traditional Korea medicine (TKM), Corni Fructus (CF) is the dry ripe sarcocarp of *Cornus officinalis* Siet. et Zucc., which is classified into the family of Cornaceae, commonly known as dogwoods. In TKM theory, CF has sour and astringent and is known to supplement the liver and kidney with its tepid properties [10]. In line with the theory of supplying the liver and kidneys, various studies have shown that CF improves liver damage in acute alcoholic liver injury and alleviates kidney damage in diabetes [11]. In addition, various pharmacological effects of CF such as reduction of oxidative stress, anti-inflammatory, and cancer cell apoptosis are known [12–15]. As active constituents of CF, loganin, morroniside, gallic acid, sweroside, cornin, etc. are known [16]. Gallic acid is a polyphenol-based compound and is well known for its high antioxidant activity; for loganin and morroniside, studies on the renal protective effect in diabetes are being actively conducted [17–21]. In addition, various effects such as apoptosis and alleviation of inflammation through the NF-*κ*B pathway are known [22, 23].

In this study, renal inflammation and fibrosis were induced through unilateral ureteral obstruction (UUO), and the renal protective effect of CF in renal fibrosis was evaluated. To elucidate the underlying mechanism of CF, which is known to supplement the kidney and inhibition of oxidative stress, we examined the NADPH oxidase and TGF-*β*1/Smad pathways in renal fibrosis.

## 2. Materials and Methods

### 2.1. Materials

2-thiobarbituric acid, phenyl methane sulfonyl fluoride (PMSF), and 1,1,3,3-tetramethoxypropane were purchased from Sigma-Aldrich (St, Louis, MO, USA). Phosphoric acid was purchased from Duksan company (Ansan, Korea). The protease inhibitor mixture solution was purchased from Wako Pure Chemical Industries, Ltd. (Osaka, Japan). The pierce BCA protein assay kit was purchased from Thermo Fisher Scientific (Waltham, MA, USA). ECL western blotting detection reagents and pure nitrocellulose membranes were purchased from GE Healthcare (Chicago, IL, USA). NOX2 (SC-130543), p22^phox^ (SC-271968), Rac1 (SC-217), LKB1 (SC-32245), p-LKB1 (SC-271924), I*κ*Bɑ (SC-1643), p-I*κ*Bɑ (SC-8404), NF-*κ*Bp65 (SC-8008), TGF-*β*1 (SC-130348), Smad2/3 (SC-8332), p-Smad2/3 (SC-11769), Smad4 (SC-7966), Smad7 (SC-365846), MMP-2 (SC-13595), TIMP-1 (SC-21734), *β*-actin (SC-47778), and histone (SC-8030) were used antibodies of Santa Cruz Biotechnology, Inc. (Dallas, TX, USA). AMPK (#2532) and p-AMPK (#2531) were used antibodies of Cell Signaling Technology, Inc. (Danvers, MA, USA). *α*-SMA (ab5694) and collagen I (ab34710) were used antibodies of Abcam (Cambridge, England). Goat anti-rabbit and goat anti-mouse immunoglobulin G (IgG) horseradish peroxidase- (HRP-) conjugated secondary antibodies were purchased from GeneTex, Inc. (Irvine, CA, USA). Zoletil®50 was purchased from Virbac Laboratory (Carros, France), and Isotroy was purchased from Troikaa Pharmaceuticals, Ltd. (Ahmedabad, India).

### 2.2. Preparation of the Plant Material

Corni Fructus (CF) was purchased from Omniherb Co., Ltd. (Daegu, Korea). A voucher herbarium specimen was verified at the College of Korean Medicine in Daegu Haany University. The herb dried Corni Fructus (100 g) was extracted by 10 times of distilled water (100°C) during 2 h. The solvent was concentrated in evaporated *in vacuo* and dried completely using a freeze dryer to obtain a powder (the yield rate of CF; 25.7%).

### 2.3. CF Analysis by HPLC Chromatogram

**Figure 1:**
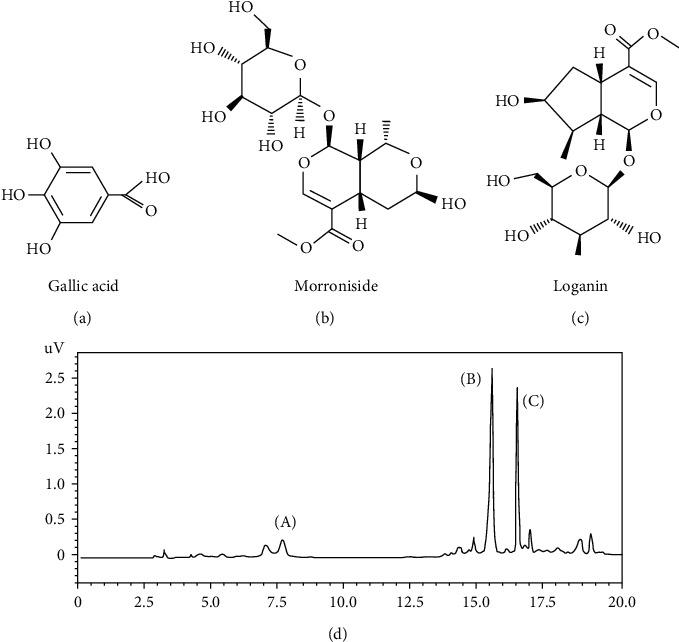
Analysis of gallic acid, morroniside, and loganin in the extract of CF. (a) Chemical structure of gallic acid. (b) Chemical structure of morroniside. (c) Loganin. (d) HPLC chromatogram of the extract of CF.

Each sample (10 mg) was dissolved in 10 mL of 50% methanol and filtered through a Dismic-13 JP membrane filter. We injected 20 *μ*L of the sample into a reverse-phase HPLC using a Phenomex Gemini NX C18 (4.6 × 150 mm, 3 *μ*m pore size), with a column temperature of 35°C. Mobile phase component was as follows: solvent A (0.1% formic acid) and solvent B (acetonitrile). The gradient conditions are as follows: A : B = 100 : 0 (0 min)⟶100 : 0 (3 min)⟶90 : 10 (5 min)⟶90 : 10 (7 min)⟶80 : 20 (12 min)⟶70 : 30 (17 min)⟶70 : 30 (22 min)⟶40 : 60 (31 min)⟶40 : 60 (35 min)⟶5 : 95 (40 min)⟶5 : 95 (43 min)⟶50 : 50 (45 min). The flow rate was 0.6 mL/min. The UV absorbance from 277 nm was monitored using an Agilent 1200 series with a multiwavelength detector (Agilent Technologies, San Jose, CA, USA). All peaks were assigned by carrying out coinjection tests with authentic samples and comparing them with the UV spectral data. The measurement was repeated three times for each sample. Representative HPLC results are illustrated in Figure 1. Quantification of gallic acid, morroniside, and loganin in the extract was performed by peak area measurement (gallic acid, 27 mg/g; morroniside, 361 mg/g; loganin, 514 mg/g).

### 2.4. Unilateral Ureteral Obstruction (UUO) Model

The animal experiments were performed according to the “Guidelines for Animal Experiment” approved by Ethics Committee of the Daegu Haany University (Approval no. DHU2021-047). The 7-week-old male Sprague-Dawley rats (body weight, 200~240 g) were purchased from DaehanBioLink (Eumseong, Korea) and used for the experiments after being adapted to environment for 1 week. Environmental conditions were set to 12 h light/dark cycle, controlled humidity (50 ± 5%), and temperature (22 ± 2°C). After 1 week adaptation, the rats were randomly divided into 4 groups (*n* = 8/group) as follows: the normal group (normal), the UUO+distilled water treatment group (control), the UUO+100 mg/kg/day CF treatment group (CF100), and the UUO+200 mg/kg/day CF treatment group (CF200). Rats were given intraperitoneal anesthesia with tiletamine and zolazepam (Zoletil®50; 37.5 mg/kg). The left ureter was isolated through a median incision and was ligated at 2 points with 2–0 silk. From the day after the operation, the drug corresponding to the each group was orally administered for 2 weeks, and on the 15th day after the operation, animals were euthanized and the kidneys were collected. Kidneys were immediately stored at -80°C.

### 2.5. Histological Examination

The kidneys were stained with three of the most suitable dyes, such as hematoxylin & eosin (H&E), Masson's trichrome (MT), and periodic acid-Schiff (PAS). The kidneys were fixed in 10% buffered formalin and then embedded in paraffin. These were sectioned with 3 *μ*m thickness (H&E) and 5 *μ*m thickness (MT and PAS). PAS-stained sections (0.5% periodic acid; 10 min, Schiff solution; 10 min) were counterstained with Harris hematoxylin for 3 min (at 25°C) and differentiated in 1% HCl-EtOH mixture, followed by blue color development in 0.5% ammonia water (30 s), dehydration in ethanol, cleaning in xylene, and examination. Each slide was observed and analyzed using the i-Solution Lite (IMT i-Solution Inc., Burnaby, BC, Canada). Quantification analysis of each positive signal for histological examination image was obtained from the randomly selected sections of at least 3 fields of each sample using Image J 1.8.0 software (U.S. National Institutes of Health, Bethesda, MD, USA).

### 2.6. Measurement of MDA Level

The malondialdehyde (MDA) level was measured according to the method of Mihara and Uchiyama [24]. 1,1,3,3,-tetramethoxypropane was used as a standard sample. After mixing sample and 1% phosphoric acid, 0.67% thiobarbituric acid was added, and boiling for 45 min at 95°C. After that, mixed butanol, and centrifuge (3,000 rpm, 10 min) to use supernatant. Dispense the supernatant, and absorbance was measured at 540 nm using a UV-VIS spectrophotometer.

### 2.7. Measurement of BUN Level

The blood urea nitrogen (BUN) level was measured using specific assay kit and a microplate fluorescence reader (Asan, Seoul, Korea).

### 2.8. Preparation of Nuclear and Cytosol Factions

The extraction of protein was performed as described by Komatsu [25]. For cytosol fractions, kidney tissues were homogenized with lysis buffer A containing 10 mM HEPES (pH 7.8), 10 mM KCl, 2 mM MgCl_2_, 1 mM DTT, 0.1 mM EDTA, 0.1 mM PMSF, and protease inhibitor mixture solution. The tissue homogenates were incubated (4°C for 30 min), and then, 10% NP-40 was mixed well. After centrifugation (12,000 rpm at 4°C for 2 min) using Eppendorf 5415R (Hamburg, Germany), the supernatant (cytosol fractions) was separated to new Eppendorf tubes. The pellets were washed twice by the lysis buffer and discard the supernatant. After that, the pellets were suspended with lysis buffer C containing 300 mM NaCl, 50 mM HEPES (pH 7.8), 50 mM KCl, 1 mM DTT, 0.1 mM PMSF, 0.1 mM EDTA, and 1% (*v*/*v*) glycerol, and protease inhibitor mixture solution was suspended and incubated (4°C for 30 min). After centrifugation (12,000 rpm at 4°C for 10 min), the supernatant (nuclear fractions) was collected in new tubes. For the estimation of NOX2/p22^phox^/Rac1/p-AMPK/AMPK/p-LKB1/LKB1/p-I*κ*B*α*/I*κ*B*α*/NF-*κ*Bp65/*α*-SMA/collagen I/MMP-2/TIMP-1/p-Smad2/3/Smad2/3/Smad4/Smad7/□-actin/histone, 12 *μ*g of proteins was electrophoresed through 8-12% sodium dodecyl sulfate polyacrylamide gel (SDS-PAGE). Separated proteins were transferred to a nitrocellulose membrane, blocked with 5% (*w*/*v*) skim milk solution for 1 h, then incubated with primary antibodies (1 : 1000), respectively, overnight at 4°C. After the blots were washed, they were incubated with anti-rabbit or anti-mouse IgG HRP-conjugated secondary antibody (1 : 3000) for 2 h at room temperature. Each antigen-antibody complex was visualized using ECL western blotting detection reagents and detected by chemiluminescence with Sensi-Q 2000 Chemidoc (Lugen Sci Co., Ltd., Gyeonggi-do, Korea). Band densities were measured using ATTO Densitograph Software (ATTO Corporation, Tokyo, Japan) and quantified as the ratio to histone or □-actin. The protein levels of the groups are expressed relative to those of the normal rat (represented as 1). We followed the methods of Lee et al. [26].

### 2.9. Statistical Analysis

Data are presented as the mean ± standard deviation. Statistical comparisons were analyzed by one-way ANOVA tests followed by the least significant difference (LSD) test using SPSS (version 26.0, IBM, Armonk, NY, USA). Values of *P* < 0.05 were considered significant.

## 3. Results

### 3.1. Renal Volume and Renal Histopathology

**Figure 2:**
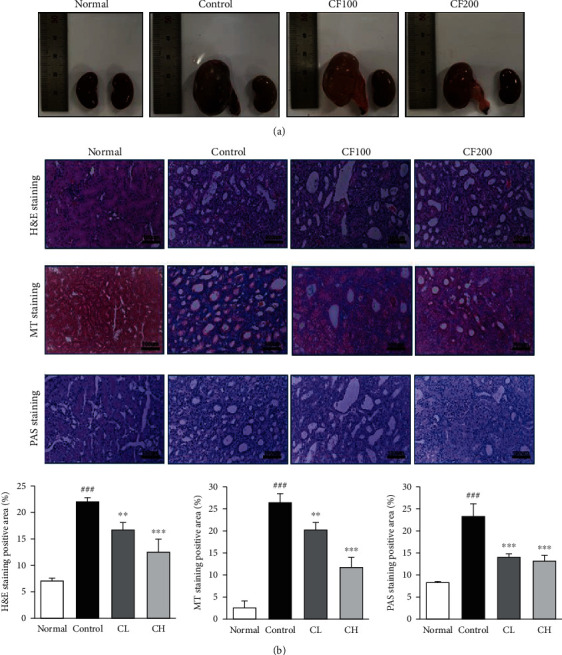
Surgical induction of unilateral ureteral obstruction and kidney histological examination. (a) Representative gross image. (b) Kidney histological examination. The scale bars represent 100 *μ*m (magnification, ×200). H&E staining; the kidneys were stained with Hematoxylin and Eosin; MT staining; the kidney were stained with Masson's trichrome; PAS staining; the kidney were stained with periodic acid-Schiff. Significance: ^###^*P* < 0.001 vs. the normal group and ^∗∗^*P* < 0.01, ^∗∗∗^*P* < 0.001 vs. the UUO control group.

When the renal volumes of the four groups were compared, it was confirmed that the renal volume increased due to ureter obstruction in the control compared to the normal, and the renal parenchyma became thinner. On the other hand, it was confirmed that the renal volume decreased in the group administered with CF, which means that fibrosis due to obstruction was successfully alleviated (Figure 2(a)).

To confirm histological changes in the kidney, tissues were examined using H&E, MT, and PAS staining. Representative images are shown in Figure 2(b). In the control compared to normal, it was confirmed that the infiltration of tubular dilatation, inflammatory cells such as neutrophil and the deposition of collagen were increased, and it was confirmed that the infiltration of inflammatory cells and the erosion of collagen were relatively decreased in the kidneys treated with CF.

### 3.2. Measurement of MDA and BUN Levels

**Figure 3:**
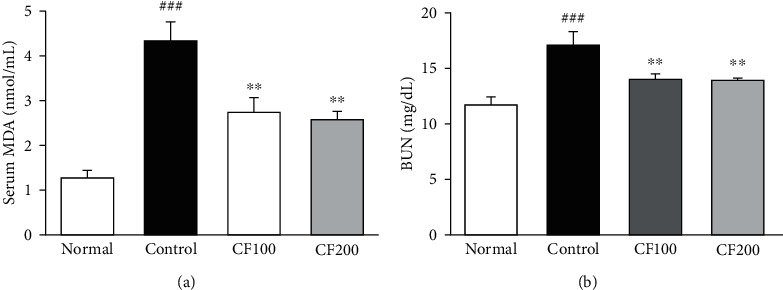
Effects of CF on MDA and BUN levels. (a) Serum MDA (nmol/mL). (b) Serum BUN (mg/dL). Bar are express as mean ± SD (*n* = 8). Significance: ^###^*P* < 0.001 vs. the normal group and ^∗∗^*P* < 0.01 vs. the UUO control group.

Oxidative stress is known to induce an inflammatory response in the body, and oxidative stress in the kidney causes kidney damage and increases the level of BUN [27].  Figure 3 shows the results of measuring MDA and BUN in serum. MDA level in the control was markedly higher compared with the normal (1.27 ± 0.17 vs.4.34 ± 0.41 mnol/mL, *P* < 0.001). MDA levels in CF100 were significantly reduced by 37% compared to the control, and in CF200, MDA level was also significantly reduced by 40%. The level of BUN, which is used as a factor of kidney damage, was also significantly higher in the control than in the normal (11.67 ± 0.66 vs.17.02 ± 1.15 mg/dL, *p* < 0.001), whereas it was confirmed that the CF-treated group significantly decreased compared to the control.

### 3.3. Expressions of NADPH Oxidase in UUO

**Figure 4:**
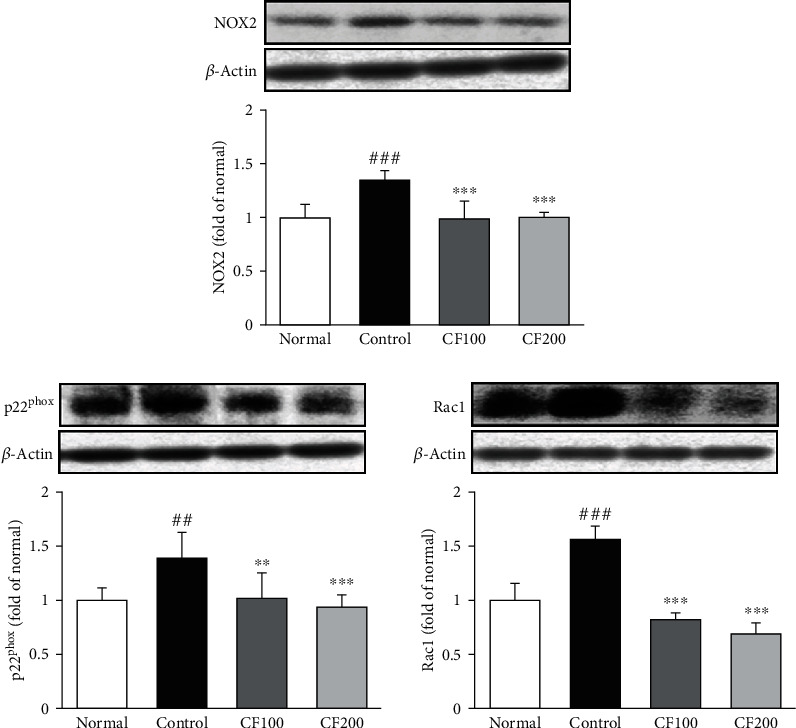
Effects of CF on NADPH oxidases. The expressions of NADPH oxidase were measured by western blotting. Bar are express as Mean ± SD (n =8). Significance: ^##^*P* <0.01, ^###^*P* <0.001 vs. the normal group and ^∗∗^*P* < 0.01,  ^∗∗∗^*P* < 0.001 vs. the UUO control group.

A number of previous studies have reported various possible connections between NADPH oxidases and oxidative stress. As shown in Figure 4, NADPH oxidases, including NOX2, p22^phox^, and Rac1, significantly increased in the UUO control group (NOX2; 1.34 ± 0.09, p22^phox^; 1.39 ± 0.24, Rac1; 1.56 ± 0.13), whereas CF treatment dramatically decreased such the increase to the level of the normal.

### 3.4. Response of Inflammation by AMPK/LKB1 and NF-*κ*Bp65 Activation in UUO

**Figure 5:**
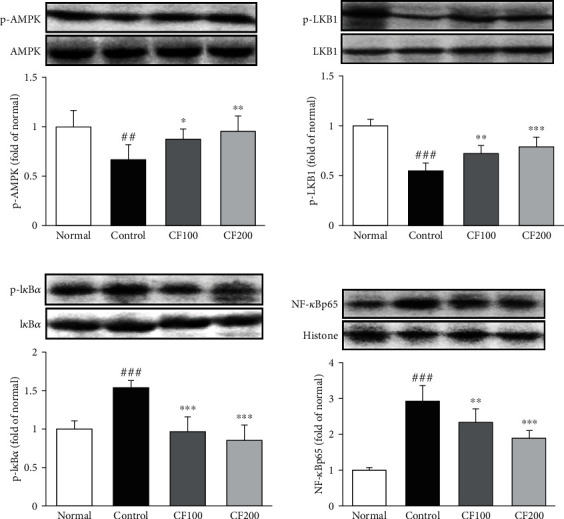
Effects of CF on AMPK/LKB1/NF-*κ*B pathway. The expressions of AMPK/LKB1/NF-*κ*B pathway were measured by western blotting. Bar are express as mean ± SD (*n* = 8). Significance: ^##^*P* < 0.01, ^###^*P* < 0.001 vs. the normal group and ^∗^*P* < 0.05, ^∗∗^*P* < 0.01, ^∗∗∗^*P* < 0.001 vs. the UUO control group.

Expressions of p-AMPK, p-LKB1, p- I*κ*B*α*, and NF-*κ*Bp65 were quantified using western blotting. As shown in Figure 5, CF-treated induced the phosphorylation of AMPK and LKB1. The phosphorylation of AMPK and LKB1 reduced the translocation of NF-*κ*Bp65 to the nucleus by inhibiting phosphorylation of I*κ*B*α*.

### 3.5. Expressions of ECM-Related Protein in UUO

**Figure 6:**
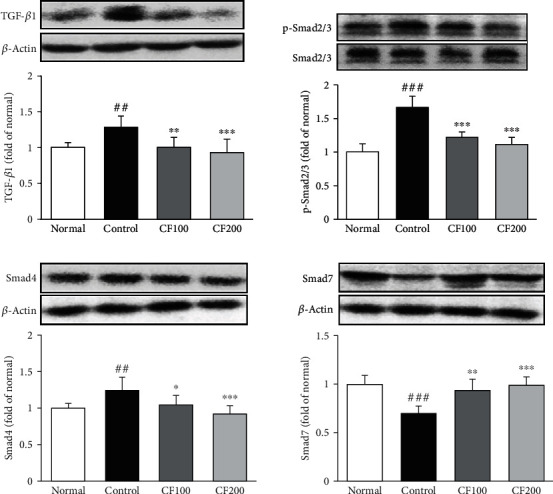
Effects of CF on TGF-*β*1/Smad signaling. The expressions of TGF-*β*1/Smad signaling were measured by western blotting. Bar are express as mean ± SD (*n* = 8). Significance: ^##^*P* < 0.01, ^###^*P* < 0.001 vs. the normal group and ^∗^*P* < 0.05, ^∗∗^*P* < 0.01, ^∗∗∗^*P* < 0.001 vs. the UUO control group.

To investigate the effects of CF on remodeling of the ECM, we performed western blot experiments of *α*-SMA, collagen I, MMP-2, and TIMP-1. Expression of ECM-related proteins is shown in Figure 6. These proteins were expressed at higher levels in the UUO control group than in the normal (*α*-SMA; 3.30 ± 0.49, collagen I; 1.84 ± 0.13, MMP-2; 1.37 ± 0.19, and TIMP-1; 1.46 ± .14). Also, CF-treated group significantly reduced these ECM-related protein levels.

### 3.6. Expressions of TGF-*β*1/Smad in UUO

**Figure 7:**
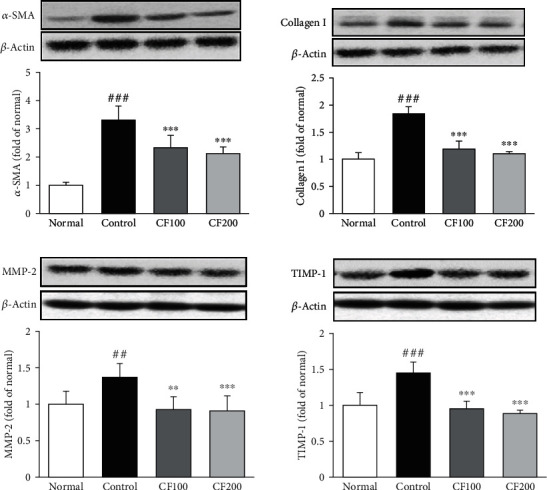
Effects of CF on extracellular matrix remodeling. The expressions of extracellular matrix remodeling were measured by western blotting. Bar are express as mean ± SD (*n* = 8). Significance: ^##^*P* < 0.01, ^###^*P* < 0.001 vs. the normal group and ^∗∗^*P* < 0.01, ^∗∗∗^*P* < 0.001 vs. the UUO control group.

We examined TGF-*β*1/Smad signaling by means of western blot. As shown in Figure 7, compared with the normal, UUO control group significantly upregulated the protein levels of TGF-*β*1 and phosphorylated Smad2/3 (TGF-*β*1; 1.28 ± 0.16 and p-Smad2/3; 1.66 ± 0.17). In addition, in the UUO control group, the expression of Samd4 was significantly upregulated and the expression of Smad7 was downregulated (Smad4; 1.25 ± 0.176 and Smad7; 0.70 ± 0.08). Compared with the UUO control group, the proteins of TGF-*β*1, p-Smad2/3, Smad4, and Smad7 were all dramatically regulated in the CF-treated group.

## 4. Discussion

Chronic kidney disease (CKD) is a disease that refers to when symptoms such as abnormal kidney function and structure persist for more than over months; due to its high mortality rate, it is considered a dangerous disease, especially for patients with a history of diabetes, hypertension, and cardiovascular disease [1]. In this study, renal inflammation and fibrosis were induced through unilateral ureteral obstruction (UUO), and the renal protective effect of Corni Fructus (CF) in renal fibrosis was evaluated. CF has been widely used in traditional Korea medicine (TKM) as a supplement for the liver and kidney [11]. In support of this theory, various studies on the renal protective effect of CF have been published [28–30]. In addition, several studies have demonstrated that the active compounds of CF, such as morroniside and logan, have renal protective effects [18, 20]. Active chemical compound of CF was detected by HPLC, and 27 mg/g of gallic acid, 361 mg/g of morroniside, and 514 mg/g of loganin were detected. These finding showed that CF may play a vital role in the improvement of the kidney. To evaluate the renal protective effect of CF, renal fibrosis was induced in the left kidney of animals. CF improved the increased renal volume and thinned renal parenchyma due to UUO and showed successful remission of fibrosis due to obstruction. Also, it was confirmed that infiltration of lymphocytes and inflammatory cells and deposition of collagen were relatively reduced in the CF-treated group compared to the UUO control group through H&E, MT, and PAS staining.

The kidney is an organ that requires a lot of energy, which makes it more vulnerable to damage caused by oxidative stress (OS) [31]. According to Khan's presentation, OS in chronic kidney disease can promote complications such as hypertension [32], and Manucha Valles reported that oxidative stress directly affects the inflammatory process and apoptosis in obstructive nephropathy [33]. Malondialdehyde (MDA), known as a factor in lipid peroxidation, contributes to OS and is known as one of the most reliable markers for determining oxidative stress [34, 35]. As a result of this experiment, the level of MDA in the serum increased by UUO was significantly reduced in the CF-treated group, and the level of BUN, known as a kidney injury biomarker, was also significantly reduced. The other production source about OS is the NADPH oxidase (NOX) family, which has a role in catalyzing the formation of peroxides from oxygen and NADPH [36–38]. Numerous studies have shown that NOX2 is associated with renal fibrosis and can alleviate renal fibrosis by inhibiting NOX2 [39, 40]. As a result of confirming the expression of NOX2, in our study, it was confirmed that the CF-treated group significantly decreased compared to the UUO control group, and the expression of p22^phox^ and Rac1 was also significantly decreased. This is thought to be involved in the alleviation of renal fibrosis in this study as in the previous study that inhibition of NOX2 alleviates renal fibrosis.

AMPK/LKB1 is a pathway that plays a key role in maintaining cellular energy homeostasis. It is known that this pathway promotes the inflammatory response by inducing NF-*κ*B activation and phosphorylation of I*κ*B*α*. Also, inflammatory response induces the synthesis of fibrosis related factors, leading to renal fibrosis [41–43]. In our study, CF significantly inhibited NF-*κ*B activation via the AMPK/LKB1 pathway, suggesting that CF negatively affects renal fibrosis progression through its anti-inflammatory effect. The extracellular matrix (ECM) is present in all tissues and regulates various functions such as proliferation, migration, and differentiation of cell. Myofibroblasts induce excessive accumulation of ECM, leading to loss of organ function and fibrosis [44–46]. In this experiment, it was confirmed that the expression of *α*-SMA and ECM-related factor (collagen I, MMP-2, and TIMP-1) was increased by UUO, and it was confirmed that the downregulation was confirmed by administration of CF. We investigated TGF-*β*1/Smad signaling, which is known to be a major regulator of renal fibrosis. Many researchers have demonstrated that TGF-*β*1 is associated with renal fibrosis. TGF-*β*1 is induced by fibroblast activation and ECM accumulation and is directly involved in the activation of Smad causing overexpression of fibrosis genes [47, 48]. Receptor-regulated Smads (R-Smad) migrate to the nucleus along with common Smad (co-Smad) to regulate the transcription of genes. In this process, inhibitory Smads (I-Smad) inhibit TGF-*β*1 signaling, reducing the formation of the R-Smad/co-Smad complex [49]. In this experiment, it was confirmed that the expression of TGF-*β*1 was suppressed by administration of CF, and the expression of Smad2/3 (R-Smads), Smad4 (co-Smad), and Smad7 (I-Smad) was regulated.

## 5. Conclusion

Taken together, CF inhibits ECM deposition and TGF-*β*1/Smad signaling by inhibiting the activity of NF-*κ*B through inhibition of oxidative stress in the UUO. We found that CF impacted renal fibrosis and inflammation in a rat model of UUO. Also, these results suggest that it could be a new material for UUO treatment.

## Data Availability

All data are contained within the article.
